# PD‐L1, PARP1, and MMRs as potential therapeutic biomarkers for neuroendocrine cervical cancer

**DOI:** 10.1002/cam4.4034

**Published:** 2021-06-02

**Authors:** Xiaoyu Ji, Lei Sui, Kejuan Song, Teng Lv, Han Zhao, Qin Yao

**Affiliations:** ^1^ Department of Obstetrics and Gynecology The Affiliated Hospital of Qingdao University Qingdao China; ^2^ Department of Pathology The Affiliated Hospital of Qingdao University Qingdao China

**Keywords:** immunotherapy, mismatch repair proteins, neuroendocrine cervical cancer, PARP1, PD‐L1, targeted therapy

## Abstract

**Objective:**

Neuroendocrine cervical cancer (NECC) is a rare cervical cancer with high aggressivity that causes poor prognosis even in the early stage. Given other neuroendocrine carcinomas and other types of cervical cancer have been proved to have expression of programmed cell death protein 1 ligand 1(PD‐L1) and poly ADP‐ribose polymerase‐1(PARP1), we would measure and analyze these proteins in this invasive cancer. The purpose of this study is to investigate the application value of PD‐1/PD‐L1 and PARP1 inhibitors in NECC.

**Methods:**

The NECC cases in our center with formalin‐fixed paraffin‐embedded tissue blocks were collected, and immunohistochemical (IHC) staining of PD‐L1, PARP1, Mismatch repair proteins (MMRs), and P53 was performed. Chi‐square test was used to analyze associations between various protein expressions. We analyzed the efficacy of immunotherapy in a recent patient with secondary recurrence after two courses of chemotherapy.

**Results:**

After rigorous screening, 20 cases were finally included. Three cases did not undergo surgical treatment because of their advanced stage. Twelve (60%) developed distant metastases or relapsed within five years, and most of them within two years. The positive rate of PD‐L1 and PARP1 were 70% and 75% respectively. Among all the cases, microsatellite instability (MSI) was seen in six cases (30%) and abnormal p53 expression was in 15 patients (75%). PD‐L1 was associated with PARP1 expression in the MSI subgroup. The patient treated with chemotherapy + VEGF inhibitor (VEGFi) + programmed cell death protein 1(PD‐1) inhibitor had an excellent improvement in clinical symptoms, tumor markers, and mass size.

**Conclusion:**

The IHC results of PD‐L1, PARP1, and MMRs suggested that NECC was the target of immunotargeted therapy. Our case confirmed that immune checkpoint therapy was effective in patients with PD‐L1 positive and MMRs loss. Considering the clinical practicability, more cases should be collected, and effective biomarkers still need to be further searched.

## Graphical abstract

The feasible immunotargeted therapy for cervical neuroendocrine tumors was discussed by studying the expression of PD‐L1, PARP1, 4 kinds of mismatch repair proteins and p53 in cervical cancer and case analysis.

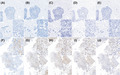

## 1 INTRODUCTION

Neuroendocrine cervical carcinoma is a rare histological type of cervical cancer, accounting for 0.9%–1.5%.^1^ Compared to common squamous cell cancer or adenocarcinoma, they are more prone to lymphatic space infiltration and lymph node involvement, as well as local and distant recurrence. But neuroendocrine carcinomas occur most frequently in the gastrointestinal tract and lungs,^2^ and the rarity of morbidity in cervix causes the limited biological and clinical data of NECC currently.

The prognosis of patients with NECC was poor, related to clinical stage closely, with a mean recurrence‐free survival of 16 months and a mean overall survival(OS) of 40 months.^3^ Given the aggressive nature of this disease, all treatment algorithms focus on multimodality treatment,^4^, ^5^ combining radical hysterectomy, systemic chemotherapy, and radiotherapy. NECC does not have a standardized chemotherapy scheme, which is mainly extrapolated from the data published in lung neuroendocrine carcinoma. The regimens particularly favored were systemic platinum or platinum and etoposide‐based chemotherapy.^3^, ^6^ Some of the clinical and pathologic features of these tumors are characteristic of the organ of origin, but other attributes are shared by neuroendocrine neoplasms irrespective of their anatomic site.^3^ Considering the particularity of its pathological type, its treatment can refer to not only the method of the general cervical cancer, but also that of other neuroendocrine cancers, like small‐cell lung cancer(SCLC),^7^ Merckle cell cancer(MCC),^8^, ^9^ neuroendocrine cancer of gastrointestinal tract, etc. In recent years, the treatment of advanced, metastatic, and recurrent cervical cancer has become difficult focus, and several clinical trials demonstrated the efficacy of immune‐checkpoint inhibitors.

Immunotherapy and targeted therapy have made some progress in present research of neuroendocrine carcinoma, including PD‐1/PD‐L1 inhibitors and PARP inhibitors (PARPi). The addition of atezolizumab to chemotherapy resulted in significantly longer OS and progression‐free survival extensive‐stage SCLC.^10^ Currently, the anti‐PD‐L1 antibody avelumab is approved for treatment of Merkel cell carcinoma.^11^ In a combined analysis of two prospective studies, Pembrolizumab was shown to be safe for use in metastatic high‐grade neuroendocrine tumors, but its efficacy as a single agent was limited.^12^ As for targeted therapy, studies have shown that PARP1 is highly expressed on mRNA and protein levels in SCLC, and SCLC was significantly sensitive to PARPi, which enhanced the efficacy of chemotherapy.^13^ Most MCC express PARP1 at high levels, and sensitivity to olaparib was observed in the MCC cell line.^14^

There is limited information on the use of immunocheckpoint inhibitors in the treatment of cervical cancer, but the applications are expanding, especially for advanced, recurrent, and metastatic cervical cancer.^15^, ^16^, ^17^ Many studies found that PD‐L1 was significantly expressed in cervical cancer,^18^, ^19^ but different evaluation criteria were used to make the results different. PARPi have been studied in a variety of cancers, including gynecologic malignancies,^20^ some clinical trials evaluated PARPi use for intractable cervical cancer are ongoing.^21^ With this background, we speculate that NECC may be an appropriate target for novel therapies such as PD‐L1inhibitors (PD‐L1i) and PARPi. We summarized the clinicopathological characteristics and follow‐up information of NECC patients in our center, and more importantly, conducted IHC expression of PD‐L1, PARP1, MMRs, and related biomarkers, to discuss the feasibility and practicability of the emerging therapies in NECC.

## 2 MATERIALS AND METHODS

### 2.1 Immunohistochemistry

**FIGURE 1:**
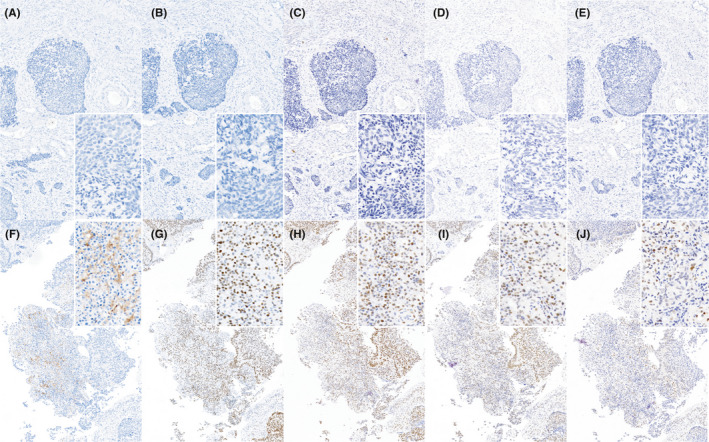
Representative images of immuno histochemical staining of NECC. Case 20: (A) PD‐L1 negative by IHC. (B) PARP1 negative by IHC. (C) MLH1 lost by IHC. (D) PMS2 lost by IHC. (E) p53 negative by IHC. Case 16: (F) PD‐L1 positive by IHC. (G) PARP1 positive by IHC. (H) MLH1 retained lost by IHC. (I) PMS2 retained by IHC. (J) p53 positive by IHC

The following IHC markers were performed: PD‐L1 (clone ab205921, Abcam), PARP1 (clone ab32138, Abcam), MLH1 (clone ab32087, Abcam), MSH2 (clone ab52266, Abcam), MSH6 (clone ab92471, Abcam), PMS2 (clone ab110638, Abcam), and p53 (clone ab1101, Abcam). The pictures viewed under a microscope are shown in Figure 1.

PD‐L1 expression was evaluated by a combined positive score (CPS). CPS is obtained by the total number of PD‐L1 positive cells, including tumor cells, lymphocytes, and macrophages, divided by the total number of living tumor cells ×100.^22^ When CPS≥1, PD‐L1 expression was positive.

PARP1 used an assessment based on degree and strength. The degree of staining was 0 (<5%), 1 (5%–25%), 2 (25%–50%), 3 (51%–75%), and 4 (>75%). Staining strength is denoted as 0 (no staining), 1 (light brown), 2 (brown), or 3 (dark brown). The total scores (ranging from 0 to 12) was multiplied two parts, which ≥3 is positive.

The four mismatched repair proteins, MLH1, MSH2, MSH6, and PMS2, were evaluated as retained (tumor cells ≥10% of any degree of staining) and loss, respectively. The microsatellite instability (MSI) was defined as any of the MMR deficiency, and all MMRs stained were defined as microsatellite stability (MSS).

The criteria to define p53 expression as “aberrant” have not been consistent among studies. In this study, the evaluation of p53 was as follows: 0: negative (no stained cells), 1: focal positive (10% stained cells); 2: patchy (11% ~ 74%); and 3: diffuse positive (75%). Scores 0 and 3 were considered as potential mutants; scores 1–2 were considered as wild‐type.^23^

### 2.2 *Case* *selection*

We identified cases diagnosed with NECC in our center over the past 10 years (January 2010–December 2019). Patients with histological data and available pathological paraffin blocks were selected. NECC is mainly diagnosed by H&E slides and IHC diagnosed by two senior pathologists. Unanimous agreement by two pathologists on the diagnosis was required for study inclusion. Patients with lack of follow‐up information were excluded. The deadline for follow‐up is 31 October 2020. Twenty cases were eventually included.

### 2.3 *Statistical* *analysis*

SPSS 25.0 software was used for inferential analysis. Correlations between the expression status of these four indicators (PD‐L1, PARP1, MSI, and p53) were determined using the chi‐squared test. A *p* value of less than 0.05 was considered statistically significant.

## 3 RESULTS

### 3.1 *Clinical* *and pathologic characteristics*

The study included 20 cases. Table 1 shows the baseline information for this cohort. The patients age ranged from 29 to 74 years (mean age 46.45, median age 44). The tumor size ranged from 1 to 12 cm (mean diameter 3 cm). Among the 20 cases, there were 14 small cell carcinomas, 1 large cell carcinoma, 1 carcinoid carcinoma, and 4 mixed carcinomas. Cases in I‐IV FIGO stages (2018) accounted for 60% (12/20), 5% (2/20), 25% (5/20), and 5% (1/20), respectively. Looking back at the case data, 13 subjects possessed HPV record and HPV negative were in only one case. In six people carried HPV type testing, 83.3% (5/6) were type 18 and 33.3% (2/6) were type 16, and one person was co‐positive for types 16, 18, and 35.

**TABLE 1:** Clinical and pathologic characteristics of our NECC cohort

id	age	stage	histology	tumor size	therapy			lymph node	Recurrence/ Progression	HPV	status	OS
					surgery	chemotherapy	radiotherapy					
1	54	IB3	S+L	4.5	Done	BEC	—	N	—		D	92
2	54	IIIC1	S	12	Done	BEC	—	P	—		A	121
3	37	IB2	S	2.5	Done	IP	—	N	—		D	57
4	41	IB2	S	2.5	Done	DP	—	N	—		A	100
5	45	IIIC2	S	1	Done	IP+EC	Done	P	Liver, bone	+	D	23
6	67	IIA1	S+A	3	Done	EC	—	N	Liver, lung	+18	D	8
7	39	IB2	S	2.2	Done	EC	—	N	Adrenal gland, pancreas	+18	D	21
8	55	IB1	S+A	1.5	Done	EC	Done	N	Recurrence		A	26
9	74	IIB	S	2.7	—	PT	Done	N	Liver	+16	D	23
10	31	IB2	S	3	Done	EC	—	N	—	+16\18\35	A	20
11	40	IB1	S	1.4	Done	EC	—	N	Lung	—	D	14
12	43	IB2	L+A	3	Done	PT	—	N	—	+	A	18
13	45	IB1	C	1	Done	—	—	N	—	+18	D	28
14	57	IB1	S	1	Done	PT	—	N	Bone, liver, adrenal gland	+	D	29
15	34	IIIC1	S	1.2	Done	EC+IP	Done	P	Peritoneal, supraclavicular	+18.	A	31
16	51	IVA	S	5	—	DP	Done	P	Liver		D	14
17	47	IB2	S	2	Done	PT	—	N	—		A	14
18	51	IIIC2	S	4	—	PT	Done	P	Retroperitoneal lymph node	+	D	15
19	35	IIIC1	S	4	Done	PT	—	P	Inguinal lymph node	+	A	55
20	29	IB1	L	2.5	Done	IP	—	N	Liver, lung, adrenal glands	+	A	53

Abbreviations: A, adenocarcinoma; A, alive; C, carcinoid; D, dead; L, large‐cell neuroendocrine carcinoma; N, negative; P, positive; S, small‐cell neuroendocrine carcinoma.

Chemotherapy regimen: BEC, bleomycin‐etoposide‐cisplatin; EC, etoposide‐cisplatin; PT, taxol + platinum; IP, irinotecan + platinum; DP, docetaxel + platinum.

After comprehensive evaluation, three patients with advanced stage did not undergo surgery and chose chemoradiotherapy. As distant metastases quickly occurred, the OS of three patients was less than two years. In the 19 patients received chemotherapy therapy, etoposide and cisplatin was the most commonly chemotherapy regimen, while paclitaxel + cisplatin,^24^ irinotecan + cisplatin,^25^ bleomycin +etoposide + cisplatin^26^ regimens were also exerted. Six patients were treated with radiation. According to postoperative pathological, there were six cases of lymph node metastasis. During the follow‐up period, 12 patients developed disease progression. The liver is the most common site of recurrence and metastasis.

### 3.2 *Interpretation* *of immunohistochemical staining*

The IHC results are shown in Table 2. Positive PD‐L1 (CPS ≥1)was observed in 70% cases (14/20) and PARP1 positive accounted for 75% cases (15/20). The MSI was defined as any of the MMR deficiency, and all MMRs stained were defined as microsatellite stability (MSS). The MSI group contained 6 cases (30%) and the MSS group contained 14 cases (70%). Among the 20 patients, 15 cases (75%) had p53 aberrant expression, in which the number of cases with scores of 0 and 3 is almost equal.

**TABLE 2:** Immunohistochemical result and HPV record of our NECC cohort

id	PD‐L1 (scores)	PD‐L1	PARP	MLH1	MSH2	MSH6	PMS2	ki67	p53	p53 (scores)	Syn	CgA	CD56
1	75	P	P	R	R	R	R	60	A	3	P	N	N
2	0	N	P	R	R	R	R	70	A	3	P	N	N
3	20	P	N	R	R	R	R	90	W	/	P	P	P
4	75	P	P	R	R	R	L	60	A	0	P	P	N
5	25	P	P	R	R	R	R	50	A	0	N	N	N
6	0	N	N	R	R	R	L	20	A	0	P	N	N
7	3	P	P	L	R	R	L	60	A	0	P	P	P
8	120	P	P	R	R	R	R	80	A	3	N	N	N
9	3	P	P	R	R	R	R	60	A	0	P	P	N
10	100	P	P	R	R	R	R	90	A	3	P	P	N
11	0	N	P	R	R	R	R	90	W	/	P	P	P
12	6	P	P	R	R	R	R	90	W	/	P	N	N
13	3	P	N	R	R	R	R	60	W	/	N	N	N
14	0	N	P	R	R	R	R	90	A	0	P	P	N
15	30	P	P	L	R	R	L	60	W	/	P	P	N
16	120	P	P	R	R	R	R	80	A	3	P	N	N
17	50	P	P	R	R	R	R	90	A	0	N	N	P
18	3	P	P	R	R	R	R	95	A	3	P	N	P
19	0	N	N	R	R	L	R	80	A	3	P	P	P
20	0	N	N	L	R	R	L	50	A	0	P	P	P

Abbreviations: A, aberrant; L, lost; N, negative; P, positive; R, retained; W, wild‐type.

In our study, no correlation was found between the results of immunohistochemical markers. Interestingly, the expression status of PD‐L1 and PARP1 in the MSI subgroup was identical (*p* = 0.004).

The diagnosis of NECC is mainly based on cell morphology combined with the IHC results of Syn, CgA, CD56, and PGP9.5,^27^ and at least an IHC indicator is positive. We mainly choosed Syn, CgA, and CD 56 as the measurement indicators, and positive rates of Syn, CgA, and CD 56 were 80%, 50%, and 35%, respectively.

### 3.3 *Description* *of the therapeutic effect of immunosuppressants in* *recurrent cases*

**FIGURE 2:**
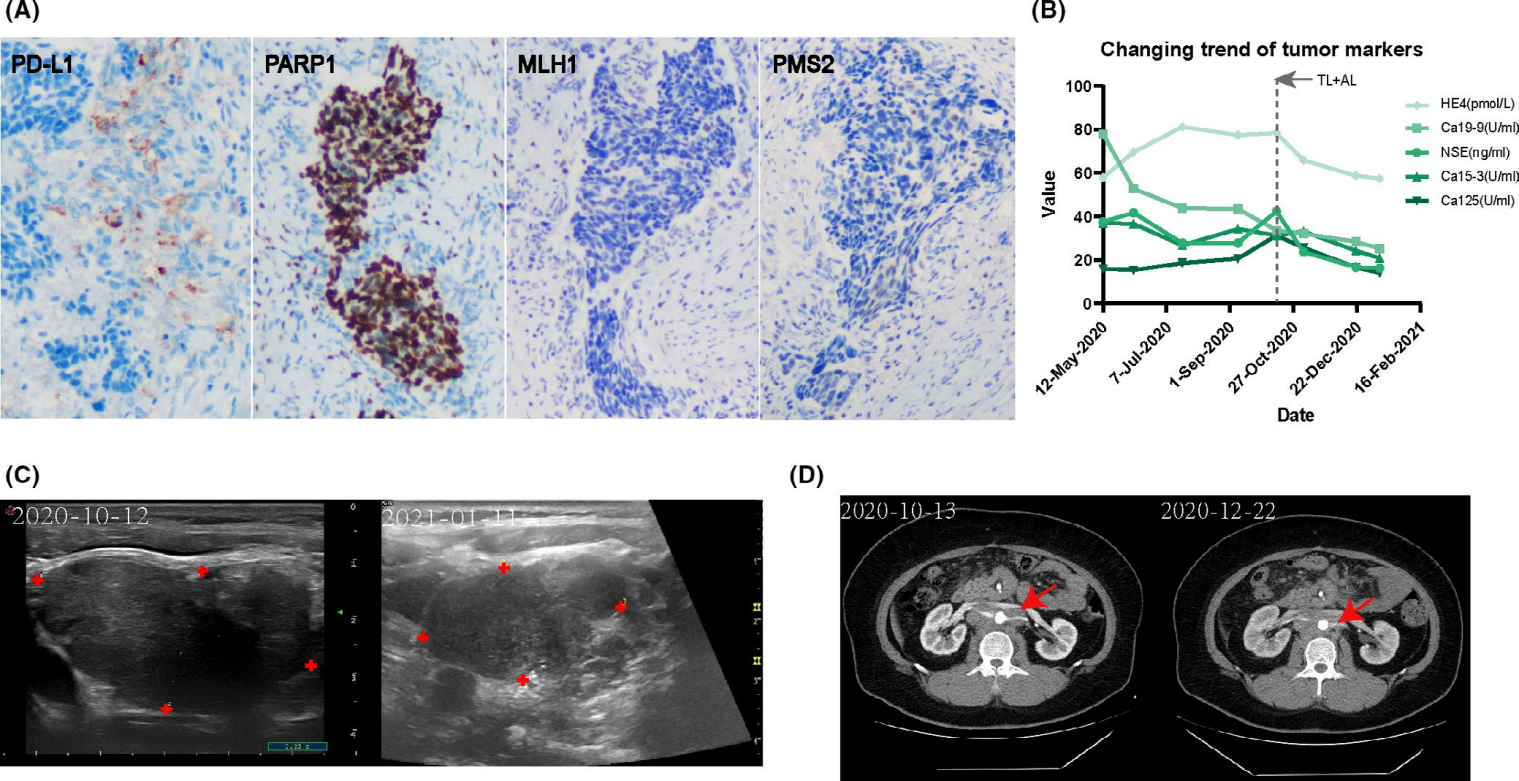
Comparison of Case 15 before and after treatment. (A) The results of immunohistochemical staining showed that both PD‐L1 and PARP1 were positive, while MLH1 and PMS2 staining were lost. (B) During the treatment, the change of tumor markers showed a downward trend, and now decreased to the normal level. The gray dotted line indicates the point in time for the addition of PD‐1 inhibitor and VEGFi. TL + AL: Tislelizumab + Anlotinib. (C) Significantly reduced left supraclavicular metastatic lymph nodes. Before treatment: 4.5*4.2*2.5cm. After treatment:3.8*3.3*1.9 cm. The red markers of cross indicate the four boundary points of the lymph nodes. (D) Contrast‐enhanced CT showed a shrinking mass near the abdominal aorta

Our case 15 was a patient with small cell cervical cancer who had a second recurrence. Etoposide/ irinotecan + cisplatin were used in the postoperative chemotherapy regimen, and 27 radiotherapy sessions were also performed. The second recurrence occurred in May 2020, showing lower abdominal pain, hematuria, and dysuria. PET‐CT results showed multiple retroperitoneal and supraclavicular lymph node metastasis. The patient's condition was in moderate response at the beginning of chemotherapy, but progressed during chemotherapy, which was mainly characterized by the increase of the level of tumor markers and the enlargement of the mass. In consideration of the PD‐L1 positive and MSI status of patients in our study (Figure 2A), PD‐1 inhibitor was added in October 2020. The treatment regimen was adjusted to 400 mg of albumin paclitaxel plus 200 mg of Tislelizumab every 3 weeks, while oral administration of Anlotinib 8 mg/day × 14 days.

At present, the patient's condition is well controlled and has the opportunity to greatly increase his progression‐free survival. First of all, the clinical manifestations related to abdominal pain and urinary system disappeared. Second, NSE, Ca199, Ca153, HE4, and other tumor markers decreased from high level to normal (Figure 2B). Finally, after the evaluation of three experienced ultrasound doctors and three radiologists, the supraclavicular lymph nodes and retroperitoneal masses were significantly reduced after 3 months of treatment (Figure 2C,D).

## 4 DISCUSSION

NECC is a rare and highly aggressive malignant tumor that was first described in 1972.^28^ The most common pathologic type of NECC is small‐cell carcinoma, which is also the type with the worst prognosis,^29^ and our statistical results show that 85% of cases have a consistent small‐cell component. The characteristics of high incidence of early lymph node and distant metastasis lead to a poorer prognosis than cervical squamous cell cancer and adenocarcinoma. Recurrence and progression occurred in 12 patients within 3 years of follow‐up, 5 of whom had lymph node metastasis at diagnosis. Currently, there is a lack of standardized treatment, mainly referring to the treatment and research experience of neuroendocrine carcinoma in other organs.^6^ The limited available therapeutic options have created an urgent demand for new treatment options. Considering the existing achievements of immunotherapy in various cancers, we decided to evaluate the application of immunotargeted therapy in NECC.

In our study, the majority of the cases had positive PD‐L1 staining, accounting for 70% of the total cases. Similar results were also found in other cohort of NECC,^30^ as well as other neuroendocrine carcinomas^31^, ^32^ also have been proved to have high expression rates. However, Cimic et al. recently reported a study of PD‐L1 and other biomarkers in which PD‐L1 was expressed at a rate of 10%.^33^ In another study of PD‐L1 and PARP1 expression of NECC, the positive expression rate of PD‐L1 was only 8%.^34^ These results are different from our results. The reason for this result may be that the evaluation criteria of PD‐L1 positive are different, and the small sample size of NECC study is also the reason for this difference that can not be ignored. The reason why we used CPS≥1 as the standard was that cervical cancer patients using PD‐L1i under this standard had a response or clinical benefit.^35^ In a study involving multiple small‐cell neuroendocrine carcinomas, RNA‐seq produced more PD‐L1 positive cases than IHC (35.7% vs. 18.5%),^36^ which implied that the actual positive cases of PD‐L1 is more than that gotten. The high expression rate of PD‐L1 in NECC indicates the great potential of PD‐L1i, at that the some neuroendocrine carcinomas were researched to admit sensitivity and effectiveness for PD‐L1 inhibitors.^10^, ^11^

The combination of PD‐L1 and MSI may be a stronger predictor of inhibitor application than PD‐L1 alone.^37^ Under the condition, the benefit rate of PD‐L1i in NECC patients increased from 70% to 85%. This benefit ratio is large enough, not to mention that data suggest that tumors with high MSS are more common than cancers with high MSI and could benefit from immunotherapy equally.^38^

Several PD‐L1/PD‐1 inhibitor combinations, including chemotherapy drugs, antiangiogenic drugs, or other immunotherapies, are also being studied. Our case 15 was a patient with secondary recurrence, and the effect was remarkable after adding immunotherapy. Chemotherapy promotes the release of a large number of antigens after the death of immunosuppressive tumor cells, which may improve the efficacy of immunotherapy.^39^ Anti‐angiogenic drugs can reduce immunosuppression, at the same time, immunotherapy can induce vascular changes or produce antivascular effects.^40^ Therefore, immunotherapy and anti‐angiogenic therapy may produce immune stimulation and vascular remodeling circulation in the tumor. At present, patients are treated with chemotherapy + PD‐1 inhibitor + VEGFi, the combined therapy strategy may achieve synergistic effect and reverse tumor immune tolerance.

The positive rate of PARP1 was 75%, and the not low percentage indicated that these patients are potential targets for PARPi. Similarly, high PARP1 expression have also been shown in other neuroendocrine cancers, and which was further proved to be sensitive to PARPi.^13^, ^14^ Among PARP1 positive cases, 80% (12 /15) were PD‐L1 positive and 20% (3 /15) were no expression. Although there was no correlation between the expression status of PD‐L1 and PARP1 (*p* = 0.272), it is obvious that the two proteins had a high positive rate and the co‐expression rate should not be ignored, and suggests that the combination of PD‐L1 inhibitor and PARPi can provide a new idea for the treatment of NECC. Based on the theory that inhibition of PARP results in the overexpression of PD‐L1 via GSK3β inactivation,^41^ University of Kentucky have developed 3 small‐molecule hybrid inhibitors of PARP and PD‐L1,and these results suggest that the properties of conjugates on tumor cells were enhanced compared with single drug.^42^ The research of PD‐L1 inhibitor, durvalumab, olaparib, or cediranib combinations are tolerable and active in recurrent womens’ cancers have entered phase II studies.^43^ Interestingly, the expression states of PD‐L1 and PARP1 in the MSI subgroup were completely consistent (*p* = 0.004),which indicate that MSI patients co‐expressed by PD‐L1 and PARP1 will benefit the most from the combination of immune checkpoint therapy and PARP‐targeted therapy. PARPi could be considered in the next treatment of case15.

P53 is one of the most frequently studied pathways in cancer. Mutations in the p53 gene can lead to the deletion or overexpression of protein expression. The high mutation rate of P53 in NECC suggests that p53‐related pathways have great research value in targeted therapy. We also found no correlation between P53 and the expression state of PD‐L1/PARP1. On the contrary, the correlation between the expression of p53 and PD‐L1 has been demonstrated in some cancers.^44^, ^45^, ^46^ P53 aberrant expression and PD‐L1 expression are closely related, which should be considered when analyzing the clinical treatment with anti‐PD1/PD‐L1 immune checkpoint inhibitors. Recent results suggest that p53 reactivation can promote innate and adaptive immunity through a variety of molecular pathways, and increase the immunogenicity of tumor cells, which provides a theoretical basis for targeted p53 drugs combined with immunotherapy.^47^

Our study was limited by the small size of the cohort, which made difficult to perform statistical analysis and search the correlation. This calls for the establishment of a NECC multicentral database to provide information for further research. In addition, all three un‐operated patients were sampled from few cervical biopsy lesions, which increased the possibility of tumor undersampling. Differences in diagnosis and management of diseases caused by the 10‐year time gap result in some incomplete data.

From what has been discussed above, NECC is a highly invasive cervical malignancy. Referring to the recent advances in the treatment of refractory cervical cancer, SCLC, MCC, etc. neuroendocrine tumors, we found that NECC may be the beneficial object of PD‐L1i and PARPi. Biomarkers are increasingly important in identifying patients who will benefit from immunocheckpoint therapy. First, the positive expression rate of PD‐L1 and PARP1 in NECC are relatively high, and co‐expression rate is high. Under the condition, the PD‐L1i and PARPi may provide a feasible treatment for NECC and may even be considered in combination. Second, MSI may be a good predictor according to other tumor studies. So the benefit ratio of PD‐L1i may be higher than expected. PD‐L1 was associated with PARP1 expression in the MSI subgroup, suggesting the specificity of MSI in immune‐targeted therapy. Thirdly, our case 15 illustrates the effectiveness of the combination of immune checkpoint therapy in the treatment of NECC. Finally, p53 mutation rate is at high levels in NECC. The indicator may estimate the malignancy of the tumor, and the relationship with prognosis deserves further exploration. In conclusion, immune checkpoint therapy and PARP targeted therapy have a great application prospect in NECC.

## 5 ETHIC STATEMENT

Our study was conducted in accordance with Good Clinical Practice guidelines and the Declaration of Helsinki (1975, revised in 2013). Prior to study commencement, the informed consent of all patients had been obtained. The clinicopathological information had been retrieved from the patients’ medical records and pathology reports. All samples were anonymized before histology and immunohistochemistry. The Institutional Review Board of Affiliated Hospital of Qingdao University considered the retrospective nature and approved the submission of our study.

## CONFLICT OF INTEREST

The authors declare no conflict of interest.

## AUTHOR CONTRIBUTIONS

Xiaoyu Ji performed data curation, formal analysis and writing draft. Lei Sui performed data curation and formal analysis. Kejuan Song and Teng Lv finished investigation and wrote the manuscript. Han Zhao analyzed the pathological features. Qin Yao reviewed and edited original manuscripts. All authors gave final approval for publication.

## Data Availability

Information about the data provided in the article is available.
